# Exploring the influence of self-perceptions on the relationship between motor competence and identity in adolescents

**DOI:** 10.1371/journal.pone.0224653

**Published:** 2019-11-04

**Authors:** Amanda Timler, Fleur McIntyre, Elizabeth Rose, Beth Hands

**Affiliations:** 1 Institute for Health Research, University of Notre Dame Australia, Perth Western Australia, Australia; 2 School of Health Sciences, University of Notre Dame Australia, Perth Western Australia, Australia; University of Warsaw, POLAND

## Abstract

**Background and aims:**

A relationship exists between an adolescent’s level of motor competence and the health of their identity. As those with low motor competence (LMC) form less healthy identities, the aim of this study was to investigate if self-perceptions mediated the negative impact of LMC on identity health.

**Methods:**

Adolescents (N = 160) completed the Adolescent Motor Competence Questionnaire (AMCQ), Assessment of Identity Development in Adolescence (AIDA) and the Self Perception Profile for Adolescence (SPPA). The mediating effect of their self-perceptions on the relationship between motor competence and identity health was examined in several ways: for the total sample, between male and females, and level of motor competence. Two motor competence groups were formed by dichotomizing their AMCQ scores (< 83 = LMC).

**Results:**

There was an indirect effect of self-perceptions of social competence, physical appearance, romantic appeal, behavioural conduct, close friendships and global self-worth on the relationship between motor competence and identity health for the total sample (N = 160, 64.4% males, M_age_ = 14.45 *SD* = .75, 12 to 16 years). No indirect effects were significant for females however close friendships and global self-worth were significant for the males. When the sample was grouped for motor competence, indirect effects of social competence, athletic competence, physical appearance, behavioural conduct, and global self-worth were significant for the high motor competence (HMC) group. The only self-perception significant for the LMC group was close friendships.

**Conclusion:**

Self-perceptions in several domains mediated the relationship between motor competence and identity health, and these differed for level of motor competence but not gender. Those with LMC who had a higher self-perception in the close friendships domain had a healthier identity. Designing physical activity programs that focus on skill development and forming close friendships are important for adolescents with LMC.

## Introduction

The development of one’s identity is a lifelong process, with the most important phase occurring during adolescence. Identity is defined as a broad spectrum of ‘who one is’ and evolves through psychosocial influences such as personal beliefs, self-awareness and self-evaluations [1, 2] as well as a range of social roles such as family, sexual, and cultural; [2, 3] outside of the self. Adolescence is a fragile phase to negotiate, as one’s identity can be impacted by many decisions, often overwhelming, about the future and can be affected by personal interests, hobbies, peer and parent support, and social media [4]. The health of an adolescent’s identity is also dependent on how they negotiate academic or occupational choices [5]. Those who develop a healthy identity have stronger personal attributes and clear future goals [3], a willingness to explore a variety of new avenues such as intimacy [6], and have a strong sense of belonging with peer groups [7]. One factor, often not considered, found to impact identity health is one’s level of motor competence [8].

Motor competence is defined as an individual’s ability to move proficiently in a range of locomotor, stability, and manipulative skills [9, 10]. An adolescent’s level of motor competence influences their involvement in a number of activities. Those with high motor competence (HMC) are motivated to participate in many age appropriate sports and recreational games [11] and consequently experience strong peer acceptance [12]. Whereas, those with low motor competence (LMC), who may also receive a diagnosis of Developmental Coordination Disorder [DCD; 13], often have poor social skills and experience more social-emotional challenges [14] such as lower social acceptance [1, 14–16]. DCD is a neuromuscular condition that reduces an individual’s ability to proficiently perform many motor tasks such as activities of daily living to the same level as their peers. The condition is evident at an early age and is not explained by another movement related disorder [13]. These difficulties may contribute to feelings of lower self-worth and higher levels of stress and anxiety [17, 18]. As a consequence, identity health is impacted as these youth do not have the same opportunities to explore their personal interests in those social settings (often team sports) that reinforce a healthy identity [3, 19]. Only recently have studies begun to report the relationship between motor competence and identity health [20, 21]. Lingam and colleagues [21] interviewed adolescents with LMC (aged 11-to 16-years) and found many formed their identity differently as they experienced greater daily struggles, challenges finding valued friendship groups and felt teachers did not understand their needs. However, those who had friends who valued their skills developed a healthier identity [21]. Therefore, there is a need to understand the role that self-perceptions play in mediating the relationship between identity health and motor competence, and whether these differ between male and female adolescents.

Harter [1] identified nine self-perception domains that described an adolescent’s competence and adequacy. These were scholastic competence, social competence, athletic competence, physical appearance, job competence, romantic appeal, behavioral conduct, close friendships, and global self-worth. Rose and colleagues [22] found that level of motor competence of 14-year-olds was associated with their self-perceptions of global self-worth, athletic competence, physical appearance, close friendships, social acceptance and romantic appeal. Those with HMC reported higher scores in a number of domains. Compared to males, females had higher self-perceptions of close friendships, with the females with HMC having higher scores for close friendships compared to those with LMC [22]. In another study, children (8-to 10-years-old) and adolescents (12-to 14-years-old) with LMC were found to have lower perceived social acceptance, athletic competence, physical appearance and global self-worth compared to the HMC group [17]. Therefore, it is plausible that self-perceptions in a range of domains may mediate the impact of an adolescent’s motor competence on their sense of identity. Those with LMC have lower self-perceptions in a number of domains [22], which may contribute to a less-healthy identity [20]. Male and female identity health may also be mediated by the strength of their self-perceptions in different domains. For example, a strong perception of close friendships may improve the relationship between a female’s identity health compared to a male’s. However to the authors’ knowledge examining how self-perceptions may mediate the relationship between an adolescent’s identity health and motor competence has not been previously considered.

Whilst researchers have independently examined motor competence in relation to self-perceptions [17, 22] and identity [21], there is limited research examining the associations between how their self-perceptions may mediate the relationship between their motor competence and identity health. The aim of this study, therefore, is to investigate this issue in a sample of adolescents and whether differences exist between males and females and those with high and low motor competence.

## Methods

### Participants

A sample of 160 (64.4% males, *M*_*age*_ = 14.44 years, *SD* = 0.75) adolescents participated in the study. They were recruited through personal contacts, an adolescent movement clinic, community sporting clubs and local schools. The inclusion criteria specified adolescents to be aged between 12-and 16-years; have English as their first language, good linguistic and cognitive ability sufficient to comprehend questions and no other diagnosed disability such as cerebral palsy or learning difficulties. The study was approved by the Human Research Ethics Committee of the University of Notre Dame in Perth, Western Australia, Department of Education and Catholic Education. This study is a part of the larger who.i.am motor competence project [8].

### Measures

**The Self-perception Profile for Adolescents** (SPPA; [23]) is a self-report questionnaire for 12-to 18-year olds and examines nine domains (scholastic, social, athletic, job competence, physical appearance, romantic appeal, behavioral conduct, close friendships, and global self-worth; [23]. It consists of 45 items, with each domain containing 5 items and 20 items reverse scored to avoid socially desirable responses. The SPPA uses a structured alternative format. The participants are given two statements to decide which one ‘is most like them’. Once they have chosen a statement they decide if it is ‘Really True for me’ or ‘Sort of True for me’ which are scored on a four point Likert scale ranging from 1 (lowest) to 4 (highest). The SPPA has evidence of internal consistency (α = 0.68~0.87) with factorial structure with an Australian sample [24].

**The Adolescent Motor Competence Questionnaire** (AMCQ; [25]) is a self-report, motor competence questionnaire for adolescents aged between 12- and 18-years. It consists of 26 items covering a range of physical skills (ball and sports) and functional tasks (activities of daily living such as dressing). It was informed by the Diagnostic and Statistical Manual of Mental Disorders (fifth edition; DSM-5) criteria for DCD [26]. Participants respond on a 4-point Likert scale of Never (1), Sometimes (2), Frequently (3), and Always (4). To account for response bias, fifteen items are reverse scored. A maximum AMCQ score is 104, with a higher score relating to higher motor competence. A score of 83 or below indicates motor difficulties may be present [25]. The questionnaire has evidence of concurrent validity against the McCarron Assessment of Neuromuscular Development [27; r = 0.49, p < 0.002}, test re-test reliability (intra-class correlation coefficients = 0.96), internal consistency (α = 0.90) and can be completed in less than 10 minutes [25]. Some example items on the AMCQ include “I can ride a bicycle” and “My hand writing is easy to read”.

**Assessment of Identity Development in Adolescence** (AIDA; [28]) is a self-report measure of identity designed for 12- to 18 years-olds. The questionnaire consists of 58 items and has a 5 point response format 0 = no (I strongly disagree), 1 = more no (I disagree), 2 = part/part (I neither agree or disagree), 3 = more yes (I agree), and 4 = yes (I strongly agree). A total identity score is calculated and ranges along a continuum from healthy (lower scores) to less healthy (higher scores). The questionnaire has evidence of construct (*r* = 0.61~0.80) and criterion validity (*d* = 2.27~2.56) and internal consistency [α = 0.73~0.94; 3]. To accommodate for cultural differences, the AIDA Australia was used in the current study with the Australian clinical cut off score greater than 148. For example, one item on the AIDA is “I often have a mental blank when I ask myself why I did things”.

### Procedures

Data collection took place over a two year period from 2014 to 2016. A total of 73 participants were recruited from sporting clubs, and 87 participants were recruited elsewhere. The questionnaire and written consent forms were distributed to personal contacts (*n* = 6), adolescent movement program (*n* = 4; screened for DCD), sporting clubs [AFL club (*n* = 140), basketball clubs (50) and a netball club (20)] and collected two weeks later (response rate 82/210 = 39%). Students in years 9, 10 and 11 attending schools in the Perth metropolitan were contacted from a list of schools generated by the primary author. A total of 34 government (137 schools in Perth = 25%), 54 Independent (62 schools in Perth = 87%) and 9 Catholic (25 schools in Perth = 36%) schools were contacted within the metropolitan area. However, only five government and seven independent schools agreed to assist in recruiting participants. Schools that opted for hard copies obtained written consent before the adolescents completed the questionnaires during an allocated class (response rate 38/65 = 58%). One independent school handed out hardcopies of the questionnaires and consent forms with a paid-reply envelope so participants could complete and place both items in the mail at a time convenient for them (response rate 30/140 = 21%). Six schools opted for the online version which enabled teachers and year group coordinators to email parents about the study (n = 19 completed). Adolescents were able to complete the questionnaire and online consent form at a time convenient to them. The overall study response rate was 39% (160/415).

### Data analysis

SPSS version 23 (IBM SPSS Inc., Chicago, IL, USA) was used to analyse the data. For some analyses, the AMCQ cut score was used to dichotomize the sample into high (>84) and low (≤83) motor competence. The AMCQ, AIDA, and SPPA scores were described using mean and standard deviation for the total sample (N = 160), males (n = 103), females (n = 57) and motor competence groups high (n = 108) and low (n = 52). Normality was assessed showing acceptable skewness (+/-1) and kurtosis (+/-1) allowing parametric tests to be used [28]. The AMCQ, AIDA, and SPPA group differences for gender and motor competence were examined using independent t-tests. Pearson’s correlations were used to examine relationships between motor competence (Total AMCQ), identity (Total AIDA) and nine self-perception domains (Mean SPPA). Self-perception domains that did not show significant correlations with AMCQ were excluded from the mediation analyses [29]. Statistical significance was set at p < .05.

**Fig 1:**
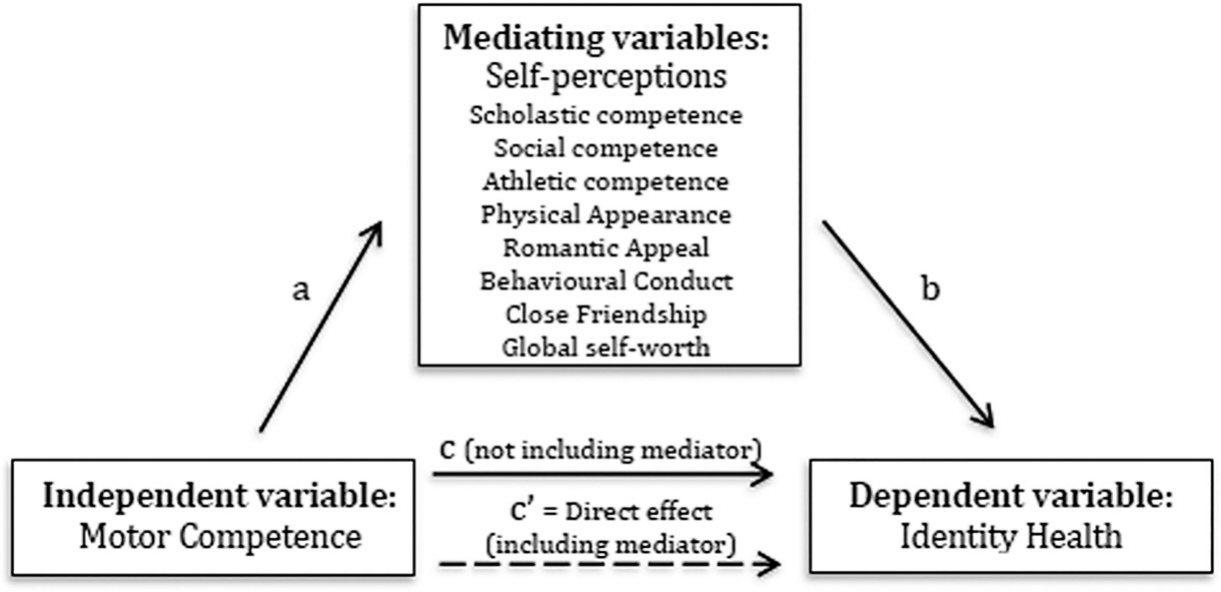
Simple mediation design showing standarised regression coefficients indicating total (c), direct (c’), and indirect effects (ab) between Motor Competence, Identity Development and eight self-perceptions with a sample of 160 adolescents.

This study used a simple mediation equation [c = c’ + ab; *a*, *b* (indirect effect), *c* (total effect), *c’* (direct effect)], adapted using regression coefficients [30] to investigate whether each self-perception variable mediated the covariance relationship between motor competence and identity health (Fig 1). The assumptions of variables being scale and normally distributed were met. To graphically depict simple mediation, the regression coefficients were entered into Medgraph (http://pavlov.psyc.vuw.ac.nz/paul-jose/medgraph/). Once the mediation was depicted, MacKinnon’s ratio [31]; indirect effect/total effect) was calculated to examine the percentage of the indirect effect on the total effect compared to the percentage of only the direct total effect (motor competence and identity health; *ab* = *c*–*c’*). In addition to the above criteria, mediation was assessed on the significance of the Sobel z-value and the 95% confidence intervals [32, 33]. The sample size (N = 160) was adequate to examine mediation (a sample size of 73 achieves 80% power to detect a change in slope from 0.30 under the null hypothesis to 0.00 under the alternative hypothesis when the standard deviation of the X's is 1.00, the standard deviation of Y is 0.90, and the two-sided significance level is 0.05). Simple mediations were examined individually for each of the eight self-perception domains for the total sample, males, females, and motor competence (high or low).

## Results

Descriptive data for the total sample, gender, HMC and LMC groups and gender and motor competence groups are reported in Table 1. It is important to note that the regression coefficients for identity are negative as a healthier identity is represented by a lower score. Overall males had a healthier identity, higher motor competence and self-perception scores in all domains except for behavioural conduct, than the females. The HMC group had a healthier identity and higher motor competence and self-perception scores in all domains except for job competence compared to the LMC group. Identity scores were less healthy (higher scores) for females and the LMC group but within a normal range. When comparing gender and motor competence, the females with LMC and HMC had less healthy identity scores compared to the males with LMC. Identity scores for the total sample ranged between 4 and 153. There were no significant differences between AMCQ scores for those completed by hardcopy (*M* = 86.98, *SD* = 10.61) compared to the online version (*M* = 83.37, *SD* = 16.20; *t* (158) = 1.30, *p* = .09), and no significant difference between AIDA scores for hardcopy (*M* = 68.33, *SD* = 33.07) compared to the online version (*M* = 67.74, *SD* = 37.42; *t* (131) = .071, *p* = .94).

**Table 1:** *Descriptive statistics [M(SD)] for the total sample*, *gender and competence level*.

		Gender		Motor competence		Gender and motor competence			
Measures	Total (N = 160)	Males (*n* = 103)	Females (*n* = 57)	HMC (*n* = 108)	LMC (*n* = 52)	HMC males (*n* = 80)	HMC females (*n* = 28)	LMC males (*n* = 23)	LMC females (*n* = 29)
Age (mths)	14.44 (.75)	14.39 (.76)	14.54 (.73)	14.47 (.77)	14.38 (.72)	14.46 (.74)	14.50 (.84)	14.13 (.76)	14.61 (.63)
Identity health (max = 232)	71.34 (34.86)	62.79 (31.09)**	**86.81 (36.21)**	64.98 (32.87)**	**84.56 (35.45)**	59.88 (31.00)*	**80.75 (33.59)**	74.35 (29.28)	92.64 (38.94)
Motor competence (max = 104)	86.55 (11.41)	89.29 (10.86)**	**81.60 (10.78)**	92.87 (4.87)**	**73.42 (9.80)**	93.46 (5.31)**	**90.54 (3.94)**	74.00 (12.00)	72.89 (7.89)
Scholastic competence	2.78 (.66)	2.87 (.63)*	**2.62 (.70)**	2.80 (.65)	2.73 (.70)	2.86 (.61)	2.64 (.75)	2.90 (.72)	2.60 (.66)
Social competence	3.04 (.58)	3.18 (.52)**	**2.80 (.62)**	3.12 (.55)*	**2.83 (.60)**	3.24 (.47)*	**2.88 (.67)**	2.97 (.62)	2.73 (.56)
Athletic competence	2.90 (.82)	3.14 (.67)**	**2.45 (.88)**	3.23 (.61)**	**2.20 (.77)**	3.34 (.53)*	**2.94 (.71)**	2.47 (.69)	1.99 (.77)
Physical appearance	2.69 (.75)	2.93 (.61)**	**2.26 (.78)**	2.77 (.69)	2.53 (.85)	2.96 (.58)	2.22 (.70)	2.83 (.74)	2.30 (.86)
Job competence	2.93 (.55)	2.94 (.54)	2.90 (.57)	2.91 (.57)	2.96 (.52)	2.94 (.53)	2.84 (.67)	2.95 (.59)*	**2.96 (.45)**
Romantic appeal	2.61 (.58)	2.73 (.52)**	**2.38 (.60)**	2.68 (.55)*	**2.45 (.61)**	2.77 (.49)*	**2.43 (.63)**	2.60 (.61)	2.33 (.59)
Behavioural conduct	3.02 (.61)	3.01 (.58)	3.04 (.66)	3.03 (.56)	2.98 (.70)	3.04 (.54)	3.01 (.62)	2.89 (.71)	3.06 (.70)
Close friendships	3.19 (.63)	3.21 (.57)**	**3.14 (.72)**	3.25 (.59)	3.06 (.67)	3.04 (.54)	3.01 (.62)	3.07 (.62)	3.04 (.72)
Global self-worth	3.05 (.63)	3.21 (.49)	2.76 (.75)	3.14 (.54)*	**2.87 (.76)**	3.26 (.55)	3.25 (.72)	3.08 (.53)*	**2.70 (.87)**

Note: HMC = High motor competence, LMC = Low motor Competence

Bold = p < .001**

P.01* (independent samples t-test)

Positive correlations between motor competence and eight self-perception domains for the total sample ranged between strong (*r* = .610, *p* < .01; athletic competence) to weak (*r* = .166, *p* = .05; behavioral conduct). Notably, the job competence (*r* = -.003, *p* .967) domain was not significant and excluded from further analyses.

### Total effect (c) between motor competence and identity

The total effect of motor competence on identity was significant with higher motor competence associated with a healthier identity (c = -.43, Standard Error [SE] = .02, *p* < .001). When analysed separately for males and females, the total effect of motor competence on identity health was significant for both males (c = -.41, Standard Error [SE] = .03, *p* < .001) and females (c = -.29, Standard Error [SE] = .04, *p* = .03). Similarly, when motor competence was analysed for high or low groups, both had the same association with identity, in that a higher motor competence was associated with a healthier identity, both the HMC total effect (c = -.40, Standard Error [SE] = .01, *p* = < .001) and LMC total effect (c = -.40, Standard Error [SE] = .04, *p* = .004) were significant.

### Mediation analysis

Fig 1 depicts the mediation pathways which were examined separately for the eight domains that reported a significant relationship with motor competence. Table 2 depicts the results from the mediation analysis for the total sample. Not all self-perception domains had a statistically significant indirect effect on motor competence and identity health. For the entire sample, social competence accounted for 23% of the indirect effect of motor competence on identity health, with the remaining 77% occurring only through the direct effect (relationship between motor competence and identity health). Similarly, the indirect effects were significant for physical appearance (20%), close friendships (23%), global self-worth (26%), romantic appeal (15%) and behavioural conduct (13%; Table 3).

**Table 2:** *Mediation analysis for the overall sample (N = 160)* .

Hypothesized mediators	Motor competence on mediator		Mediator on Identity health		Motor competence on Identity health		Mediated effect		
	a (SE)	*p*	b (SE)	*p*	c‘ (SE)	*p*	ab	95% CI	Sobel *p*
Scholastic competence	**.17 (.01)**	**.034**	**-.33 (3.59)**	**< .001**	**-.37 (.21)**	**< .001**	-.39	-.36 to .01	.065
Social competence	**.30 (.00)**	**< .001**	**-.33 (4.26)**	**< .001**	**-.33 (.22)**	**< .001**	**-.43**	**-.52 to -.11**	**.003**
Athletic competence	**.61 (.01)**	**< .001**	-.11 (3.85)	.226	**-.36 (.28)**	**< .001**	-.33	0 to 0	.284
Physical appearance	**.22 (.01)**	**.005**	**-.54 (2.79)**	**< .001**	**-.31 (.18)**	**< .001**	**-.61**	**-.64 to -.12**	**.004**
Romantic appeal	**.21 (.00)**	**.007**	**-.25 (4.31)**	**.001**	**-.38 (.22)**	**< .001**	**-.33**	**-.32 to -.02**	**.030**
Behavioural conduct	**.17 (.00)**	**.035**	**-.34 (3.91)**	**< .001**	**-.37 (.21)**	**< .001**	**-.40**	**-.34 to -.01**	**.040**
Close friendships	**.30 (.00)**	**< .001**	**-.42 (3.78)**	**< .001**	**-.30 (.21)**	**< .001**	**-.51**	**-.62 to -.18**	**< .001**
Global self-worth	**.30 (.00)**	**< .001**	**-.57 (3.31)**	**< .001**	**-.26 (.18)**	**< .001**	**-.65**	**-.81 to -.27**	**< .001**

Bold indicates statistical significance of p < .05

a = standardised regression coefficient of Motor competence on mediator

ab = product-of-coefficients

b = standardised regression coefficient of the changes in hypothesized mediator and changes in Identity health

c’ = standardised regression coefficient of Motor competence predicting Identity health with adjustment for mediator

**Table 3:** *Mediation analysis for males (n = 103)*, *and females (n = 57)*.

Hypothesized mediators	Motor competence on mediator		Mediator on Identity health		Motor competence on Identity health		Mediated effect		
	a (SE)	*p*	b (SE)	*p*	c‘ (SE)	*p*	ab	95% CI	Sobel *p*
**Males**									
Scholastic competence	.09 (.01)	.367	**-.27 (4.33)**	**.003**	**-.39 (.25)**	**< .001**	-.30	-.23 to .10	.422
Social competence	**.24 (.01)**	**.016**	**-.24 (5.48)**	**.010**	**-.35 (.26)**	**< .001**	-.32	-.34 to .03	.092
Athletic competence	**.55 (.01)**	**< .001**	-.13 (5.01)	.243	**-.34 (.31)**	**.002**	-.32	-.54 to .14	.247
Physical appearance	.12 (.01)	.238	**-.48 (3.99)**	**< .001**	**-.35 (.23)**	**< .001**	-.52	-.46 to .12	.252
Romantic appeal	**.22 (.01)**	**.028**	**-.37 (5.13)**	**< .001**	**-.33 (.25)**	**< .001**	-.44	-.46 to .08	.070
Behavioural conduct	.18 (.00)	.071	**-.38 (4.55)**	**< .001**	**-.34 (.24)**	**< .001**	-.44	-.42 to .02	.067
**Close friendships**	**.33 (.01)**	**.001**	**-.36 (4.90)**	**< .001**	**-.29 (.26)**	**.002**	**-.46**	**-.59 to -.08**	**.009**
**Global self-worth**	**.23 (.00)**	**.021**	**-.45 (5.17)**	**< .001**	**-.31 (.24)**	**< .001**	**-.53**	**-.53 to -.04**	**.023**
**Females**									
Scholastic competence	.16 (.01)	.222	**-.40 (6.23)**	**.002**	-.22 (.41)	.072	-.44	-.62 to .16	.251
Social competence	.21 (.01)	.111	**-.38 (7.21)**	**.004**	-.21 (.41)	.099	-.42	-.65 to .12	.179
Athletic competence	**.57 (.01)**	**< .001**	.06 (6.54)	.728	**-.32 (.53)**	**.049**	-.13	-.49 to .70	.728
Physical appearance	.07 (.01)	.536	**-.58 (4.84)**	**< .001**	**-.25 (.35)**	**.022**	-.60	-.66 to .30	.618
Romantic appeal	-.00 (.00)	.986	-.02 (7.82)	.902	**-.29 (.44)**	**.032**	-.02	-.02 to .02	1.00
Behavioural conduct	.19 (.01)	.151	**-.38 (6.73)**	**.003**	-.21 (.41)	.086	-.42	-.61 to .11	.177
Close friendships	**.27 (.01)**	**.043**	**-.57 (5.57)**	**< .001**	-.13 (.37)	.233	-.61	0 to .03	.062
Global self-worth	.22 (.01)	.099	**-.68 (4.66)**	**< .001**	-.14 (.33)	.161	-.71	-.96 to .10	.111

Bold indicates statistical significance of p < .05

a = standardised regression coefficient of Motor competence on mediator

ab = product-of-coefficients

b = standardised regression coefficient of the changes in hypothesized mediator and changes in Identity health

c’ = standardised regression coefficient of Motor competence predicting Identity health with adjustment for mediator

### Gender differences in mediating self-perceptions

Two self-perception domains showed an indirect effect on the relationship between motor competence and identity health for males, these were close friendships (30%) and global self-worth (19%). No self-perception domains had an indirect effect on this relationship for the females even though the direct effect was significant (Table 3).

### Motor competence differences in mediating self-perceptions

Differences in effects for several self-perception domains were identified between the HMC and LMC groups. In the HMC group, 26% of the physical appearance domain showed an indirect effect, with the remaining 74% occurring only through the direct effect. A similar effect was significant for global self-worth (30%), social competence (18%) and behavioural conduct (23%) domains (Table 4). For the LMC group only close friendships (40%) showed a significant effect (Table 4).

**Table 4:** *Mediation analysis for the HMC (n = 108) and LMC (n = 52) groups*.

Hypothesized mediators	Motor competence on mediator		Mediator on Identity health		Motor competence on Identity health		Mediated effect		
	a (SE)	*p*	b (SE)	*p*	c‘ (SE)	*p*	ab	95% CI	Sobel *p*
**HMC**									
Scholastic competence	**.31 (.01)**	**.001**	**-.19 (4.66)**	**.041**	**-.34 (.62)**	**< .001**	-.29	-.83 to .04	.077
**Social competence**	**.21 (.01)**	**.029**	**-.45 (4.82)**	**< .001**	**-.30 (.54)**	**< .001**	**-.51**	**0 to -.02**	**.042**
**Athletic competence**	**.30 (.01)**	**.002**	**-.22 (4.97)**	**.018**	**-.33 (.62)**	**< .001**	**-.32**	**-.90 to .01**	**.057**
**Physical appearance**	**.30 (.01)**	**.002**	**-.54 (3.70)**	**< .001**	**-.23 (.52)**	**.003**	**-.61**	**0 to -.36**	**.003**
Romantic appeal	.21 (.01)	.207	**-.38 (4.97)**	**< .001**	**-.35 (.56)**	**< .001**	-.42	-.83 to .19	.220
**Behavioural conduct**	**.33 (.01)**	**.001**	**-.29 (5.36)**	**.002**	**-.30 (.61)**	**.001**	**-.39**	**0 to -.12**	**.016**
Close friendships	.16 (.01)	.109	**-.46 (4.38)**	**< .001**	**-.32 (.53)**	**< .001**	-.51	0 to .14	.127
**Global self-worth**	**.32 (.01)**	**.001**	**-.46 (5.02)**	**< .001**	**-.25 (.56)**	**.003**	**-.54**	**0 to -.33**	**.003**
**LMC**									
Scholastic competence	.16 (.01)	.266	**-.52 (5.60)**	**< .001**	**-.32 (.40)**	**.006**	-.57	-.83 to .24	.284
Social competence	.16 (.01)	.251	**-.45 (4.82)**	**< .001**	**-.30 (.54)**	**< .001**	-.17	-.25 to .12	.501
Athletic competence	**.28 (.01)**	**.041**	-.03 (6.38)	.821	**-.41 (.50)**	**.005**	-.08	-.24 to .30	.821
Physical appearance	.09 (.01)	.525	**-.56 (4.41)**	**< .001**	**-.35 (.38)**	**.002**	-.59	-.74 to .27	.508
Romantic appeal	.10 (.01)	.480	**-.04 (7.71)**	**.004**	**-.39 (.48)**	**.004**	-.08	-.11 to .09	.785
Behavioural conduct	.16 (.01)	.263	**-.39 (6.09)**	.003	-.34 (.44)	.008	-.44	-.62 to .19	.299
**Close friendships**	**.47 (.01)**	**.015**	**-.37 (7.34)**	**.011**	**-.22 (.50)**	**.117**	**-.48**	**0 to -.05**	**.033**
Global self-worth	.19 (.01)	.178	**-.73 (3.83)**	**< .001**	**-.26 (.30)**	**.003**	-.78	0 to .23	.178

**Bold indicates statistical significance of p <** .**05**

a = standardised regression coefficient of Motor competence on mediator

ab = product-of-coefficients

b = standardised regression coefficient of the changes in hypothesized mediator and changes in Identity health

c’ = standardised regression coefficient of Motor competence predicting Identity health with adjustment for mediator.

## Discussion

Among adolescents, the relationship between motor competence and identity is mediated by a number of self-perceptions. When the data were separated for males and females and high and low motor competence clear differences emerged. More self-perception domains influenced the relationship between motor competence and identity heath among those with HMC. The self-perceptions of close friendships and global self-worth strengthened this relationship for males. Only higher perceptions of close friendships lead to a healthier identity for those with LMC. In other words, if they had a strong group of close friends, the potentially negative impact of LMC was less likely to damage their identity health. Some of these results are surprising and could be explained in a number of ways.

Having a positive perception of one’s physical appearance includes looking presentable and being satisfied with one’s body. This was particularly strong for the HMC group, and contributed to the relationship between a healthier identity and higher motor competence. It is likely those with HMC value the physical domain, and therefore feeling attractive would be affected by their level of fitness, ability to participate at a high level in physical activity and sports and body satisfaction [1]. Body satisfaction is based on an individual’s ability to build muscle bulk (males) and be toned and slim [females; 1, 4]. Physical appearance did not mediate the relationship between motor competence and identity health for males, females or those with LMC in this study. During adolescence, one way to achieve these ideal body types requires physical activity and a higher level of motor competence. This may lead to a more positive sense of self and identity for the HMC group, as those who feel good inside [3] are more likely to portray their physical attractiveness towards others [1]. Vedul-Khelsas and colleagues [34] found physical fitness and motor competence among 11-to 12-year-olds was positively correlated with physical appearance as well as perceptions of social competence, athletic competence, and general self-worth. Therefore feeling physically attractive may also attribute to positive perceptions in other domains and lead to a healthier identity. Similarly, one’s perception of romantic appeal involves being romantically interested, feeling attractive, and enjoying dating [35] which contributed to a healthier identity for all adolescents in this study. Kerpelman et al. [36] found that regardless of an adolescent’s relationship status (in a romantic relationship or not having a history of dating) they valued intimacy as important for their identity health.

The social competence domain relates to the ability to make friends and having good social skills and understanding social cues [1]. Social competence was important for the HMC group and may be closely related to the derived benefits of being physically active. Popularity and feeling socially accepted during adolescence is often influenced by participating in a range of age appropriate activities such as team sports [18, 37] or school dances. Ryska [38] found that sport participation during high school, led to the development of positive socialisation habits and higher competence in other domains such as scholastic competence, behavioural conduct, physical appearance and a healthy self-concept. The feeling of being socially competent may contribute to the development of a healthy identity as peer support develops a sense of belonging [3] and confidence within the self [1]. The importance of forming relationships through play from childhood to adolescence assists in the development of emotional, social and cognitive skills which contributes to a positive sense of self and identity [39]. The social competence domain did not positively influence the association between identity health and motor competence for the LMC adolescents. Those with LMC often experience higher levels of anxiety when trying to build social networks and feel socially isolated [17]. Lower social support may also be a consequence of less involvement in physical activities [40] and greater participation in sedentary, anti-social activities such as watching television.

The self–perception domain of behavioural conduct refers to the ability to avoid trouble making, feeling good about individual actions and doing the right thing [35]. Behavioral conduct was associated with motor competence and identity health for those with HMC. Perceptions of ones’ ability to behave well may contribute to identity health for this group given their ease of participating in social situations, the ability to read social cues and respect significant others [3], including their parents [41], which together help to develop lasting relationships. Ryska [38] found that young athletes with high levels of task motivation had higher self-perceptions in behavioral conduct, scholastic competence and physical appearance. Adolescents who are involved in physical activities and sports, mostly those with HMC, develop good sportsmanship, ability to resolve conflicts, and how to win and lose graciously, all examples of good behaviour [1, 3].

The close friendships domain involves the development of close and intimate friendship groups. This emerged as an important mediator for the males and those with LMC. Peer friendships affect an adolescent’s self-reported happiness [42], develop a secure attachment to friendship groups and reduce loneliness [43]. Males tend to socialise in large groups and develop close friendships through their participation in many physical activities regardless of motor skill competence [15, 44] more so than females. Doumen and colleagues [43] also found positive friendships were related to a healthier identity whereas those with a more diffused (less-healthy) identity experienced more loneliness.

Interestingly, close friendships was the only mediator important for those with LMC who are often socially isolated [16], as they face greater social-emotional difficulties, peer victimization; and internalizing symptoms [17, 40]. In this study, those with LMC, who had a higher self-perception of close friends had a healthier identity. Brewer and Gardner [45] suggest adolescents who are viewed as different are more likely to be excluded from valuable peer group experiences. Skinner and Piek [17] found those with LMC often withdraw from social situations to protect their self-esteem and experienced greater levels of anxiety. Interviews with adolescents with LMC revealed that supportive environments, including transport to recreational centers and parent encouragement increased their physical activity participation [46]. Social support may also improve the school performance of those with LMC as peers help clarify classroom instructions [16] and provide a sense of belonging which empowers their sense of identity [21]. Other than close friendship, no other self-perception domians, mediated the relationship between identity health and motor competence for the LMC group. Those with LMC form their identity differently to their peers as they overcome a greater number of difficulties within the home and school [20], and often experience poorer mental health [47] which may suggest they have fewer positive domains to draw from to form a healthy identity. Fitzpatrick and Watkinson [48] also found those with LMC avoided activities that led to humiliation and embarrassment such as participating in games and worried more about past experiences. Therefore, they experience greater stresses and difficulties, and fewer opportunities to develop resiliency skills [49] leading to repeated failures in a number of domains. This may explain why the close friendships domain was the only one to contribute to a healthier identity and reduced the negative impact of LMC.

For the HMC group, the close friendship domain did not have a strong mediating influence. Those with HMC participate in many physical activities that involve social opportunities, experience greater social acceptance and inclusion [15, 37]. They develop more confidence and opportunities to participate, build larger social networks, and do not depend on their sporting activities to develop their social networks. They build their friendship groups from many areas [24, 42].

Global self-worth is defined by Harter [1] as feeling good and happy, liking personal life goals and academic or employment outcomes [35] and was influential for the HMC group and males. For the HMC group and males, participation and successful involvement in physical activities is highly valued and may contribute to the health of their identity as they experience greater social acceptance [37] which contributes to their perception of global self-worth [1] in comparison to those with LMC who are less motivated to engage in sports and have lower perceptions of global self-worth [11, 24, 50]. A perception of global self-worth did not mediate the relationship between motor competence and identity health among the females. Female adolescents often experience greater fluctuations in their self-esteem and self-confidence [4] compared to male adolescents who have higher self-perceptions in a range of domains, including global self-worth [22, 23]. A females sense of self changes to a great extent than males, may explain why general worth did not positively influence this relationship.

Scholastic competence was the only self-perception domain that did not mediate the relationship between identity health and motor competence. This domain is related to doing well at school and achieving high grades [1] and is reflective of personal achievements and willingness to complete tasks. Therefore the link between motor competence and academic achievement may be tenuous in this instance. Tatlow-Golden and Guerin [51] found that school based competencies were less important for an adolescents identity and self-concept as they placed greater importance on peer-based activities such as skateboarding and playing video games to develop their sense of self.

Overall, no self-perception domains mediated the relationship between motor competence and identity health for the females. It was surprising that close friendships was not significant, as other studies have found many females derive happiness from their friendships [24, 42] and perceive close friends as important for this sense of self regardless of motor competence [24]. Bédard, Bouffard, and Pansu [52] found that females perceived greater social-emotional susceptibility (self-evaluations, depressive symptoms, social anxiety, and associated with social avoidance) regarding peer support, compared to males. Female adolescents also respond more to peer related feedback than males [20]. This may be due to females’ placing less importance on their motor competence, as only individual self-perceptions of athletic competence (*p* < .001) and close friendships (*p* = .043) were hypothesized indirect effects (a) on a female’s motor competence, but neither did not remain significant mediators and showed no direct effect in this study (Table 3). Females may place greater importance on self-image and body satisfaction [35] and develop their identity through different experiences such as creative activities [4], or going shopping [4]. A female’s identity health be far more complex and interactive than just from singular domains. Therefore, the self-perception subdomains that are important for female adolescents and their identity health may not be reflected in this study. Today, social media usage among females relates to higher appearance-related feedback, body dissatisfaction, emotional symptoms and self-preservation compared to males [53]. During adolescence, females are less motivated to participate in physical activities and sports, or prefer to participate in more flexibility skills such as dancing [52, 54] compared to males. Haugen, Ommundsen, and Seiler [55] also found that physical fitness measures did not predict self-perceptions of physical appearance for female adolescents. This may lead to females devaluing their motor competence level when it comes to their sense of identity.

### Limitations and recommendations for future research

The sample size was adequate, although generalization to the broader Australian population is not possible due to the limited recruitment sources. As expected, significant differences in motor competence scores were found between those recruited through sporting clubs (*M* = 91.75, *SD* = 6.95) compared to those recruited elsewhere (*M* = 82.18, *SD* = 12.57; *t* (138.15) = 6.08, *p* < .001). The latter group comprised adolescents from the community and attending a movement enhancement clinic. One consequence was fewer females than males participated in the study. Hardcopy and online questionnaires were used to reach an adequate sample size, although no significant differences between hardcopy and online versions were found. A number of methods exist for testing mediation such as regression coefficients or Structural Equation Modeling. Simple mediation using regression coefficients was used in the current study to accommodate for multiple comparisons.

Although studies have examined motor competence, self-perceptions and identity independently, this is the first study to examine these three variables. This is a strength of this study as clear associations for different mediators were seen for males, HMC and LMC groups. This study demonstrates the importance that individual self-perceptions can have on identity. Further longitudinal studies would benefit the understanding of how self-perceptions mediate motor competence and identity across adolescence, which may allow further gender differences to be explored.

## Conclusions

The relationship between motor competence and identity health is influenced by a person’s self-perceptions in a range of domains. The impact of these differ according to an adolescent’s level of motor competence and gender. Those with HMC had higher self-perception scores in a number of domains. Perceptions of close friendships appear important for those with LMC and males, whereas perception of global self-worth was important for HMC group and males. No associations were found for adolescent females in this sample which could indicate that their identity may be more strongly influenced by different experiences. These findings are important as they highlight how self-perceptions may impact the health of identity, particularly for those with LMC. These findings suggest that movement based interventions should consider the development of positive self-perceptions using strategies that focus on particular domains identified in this study. For example, those with LMC could participate in physical activity programs that focus on skill development and forming close friendships during physical activity participation.
